# Impact of Maternal Age on the Repairing Capacity of Oocytes on Paternal DNA Damage

**DOI:** 10.1007/s43032-025-01911-w

**Published:** 2025-06-12

**Authors:** Seda Karabulut, Pelin Kutlu, Oya Korkmaz, Lima Oria

**Affiliations:** 1 International School of Medicine, Department of Histology and Embryology, Istanbul Medipol University, Istanbul, Turkey; 2 Institute of Health Sciences, Histology and Embryology, Istanbul Medipol University, Istanbul, Turkey; 3 School of Medicine, Department of Histology and Embryology, Malatya Turgut Ozal University, Malatya, Turkey; 4 International School of Medicine, Istanbul Medipol University, Istanbul, Turkey

**Keywords:** Infertility, Maternal age, Sperm DNA fragmentation, ICSI, DNA repair

## Abstract

Sperm DNA fragmentation is a primary cause of male infertility, affecting ART outcomes and success rates. Sperm has no DNA repair mechanisms because it is transcriptionally quiet. Oocytes’ repairing capacity is one of the suggested methods to overcome the situation. However, based on the hypothesis that oocyte age may also have an impact on this talent, we aimed to reveal the role of female age in repairing sperm DNA damage in our study. A total of 540 couples undergoing ICSI were grouped based on the maternal age, and the groups were further divided into two groups based on the severity of DNA damage rates, as the low (< 30% SDF) and high DNA fragmentation (≥ 30% SDF). Laboratory and clinical outcomes were compared between the groups. No significant differences were observed in younger patients (≤ 36 y) and patients between 37–40 years. However, when maternal age was > 40, significantly lower embryo and blastocyst quality, blastocyst development, pregnancy, and implantation rates were observed. Our findings suggest that older oocytes may have a reduced ability to repair sperm DNA damage, as demonstrated by poorer IVF outcomes of couples with higher SDF scores and advanced maternal age (≥ 40).

## Introduction

Infertility is defined as the inability of a couple of reproductive age to conceive following 12 consecutive months of regular, unprotected, sexual intercourse [1]. The condition is a prevalent health issue among couples, with an average of 8–15%. Male factors account for ~ 50% of all infertility cases [2–5]. In vitro fertilization (IVF) techniques are commonly used therapeutic approaches, however, the overall success rate cannot exceed 40%. To diagnose male infertility, semen analysis is a routinely used diagnostic method, which is reported not to reflect the overall fertility status of a man. Recently, additional parameters not diagnosed by classical semen analysis were reported to contribute to the male fertility potential [6], one of which is the DNA status of sperm [7]. DNA damage (fragmentation) is reported to be associated significantly with the reproductive potential of males [8], which contributes to 25–80% of all infertility cases, as well as increasing the risk of genetic diseases in the offspring [9, 10]. DNA damage is reported to affect fertilization, implantation rates, and thus the overall IVF success rates, and the first visible effect is suggested to be observed on the 3rd day of embryonic development when the embryonic genome becomes activated [10, 11]. The proposed reason is that the genomic activation of sperm occurs later than the oocyte because of its tighter chromatin packaging structure [12]. Human sperm DNA is packaged with protamine proteins instead of histones to fit in a small amount of cell cytoplasm. Small cytoplasmic volume enables the sperm to move faster and quicker and thus reach the oocyte, which is its primary task. However, it causes the cell to be transcriptionally less active/silent, which lacks the factors required for other metabolic functions, such as DNA repair mechanisms [13].

Women develop their oocytes during fetal life, and are born with all the oocytes they will ever have throughout their lives, ensuring the best potential oocytes survive and the damaged ones are eliminated until reproductive age. Unlike females, male spermatogenesis continues throughout men’s lives, thus increasing the tendency for DNA damage [14].

The lack/limited capacity of the DNA repair mechanisms is of great importance for gametes because it has a high potential to affect the offspring’s health [15]. Apoptotic mechanism is one of the mechanisms used to eliminate unhealthy sperm cells with damaged DNA during spermatogenesis [15]. However, it is demonstrated that the sperm cells can either be rescued from apoptosis or demonstrate no visible signs of abnormality, although they undergo apoptosis [16]. This may lead to problems in IVF where natural sperm selection mechanisms are bypassed because a single sperm is used randomly with a higher risk of carrying damaged DNA [15]. The situation worsens if the DNA fragmentation rate is higher, which is known to be higher in infertile couples, compared with fertile patients [7, 17]. In infertile cases, a DNA fragmentation rate of ≥ 30 is reported to adversely affect the IVF outcome parameters [18].

Another proposed mechanism to solve the DNA damage problem is repairment by another cell, -the oocytes-, as soon as it enters the oocyte cytoplasm during fertilization [15]. The oocytes are transcriptionally active cells and house the DNA repair mechanisms [19]. The safeguarding role of oocytes is suggested to protect the offspring from genetic mutations [20] since the oocyte is the only gamete that can repair itself and the incoming sperm cell. Oocytes are suggested to have some capacity to repair sperm DNA damage, and oocyte age is suggested to be a factor in determining this capacity [21], however, the issue remains unclear [22, 23].

Some of the proposed mechanisms include age-related decline of BRCA-related ATM-mediated DNA repair pathway [24], DNA base-excision repair (BER) pathway proteins, including APE1, PARP1, XRCC1, Polβ (POLB), and LIG3, which is responsible for single-strand DNA (ssDNA) repair [25].

The objective of this study was to investigate the role of maternal age in reducing the sperm-born negative effects of SDF on ICSI outcomes.

## Materials and Methods

### Study Design

The present study was conducted in accordance with the ethical standards of the Declaration of Helsinki and national and international guidelines. All participants were informed, and their written consent and signatures were obtained.

This retrospective cohort study was conducted on infertile couples who underwent an ICSI cycle in İstanbul Okan Hospital, ART Center, Turkiye, from February 2019 to September 2023. Patient’s and cycle data were obtained through the medical record system of the IVF center.

Each patient underwent a thorough medical history check and a routine physical, hormonal, and genetic examinations. The inclusion criteria for the female partner were as follows: Body mass index (BMI) < 30 kg/m^2^, a baseline follicle-stimulating hormone (FSH) level of < 10 IU/L. The male partners diagnosed with oligoasthenoteratospermia (OAT) were included in the study. Males were excluded if they had cryptorchidism, genetic abnormality, varicoceles, hypogonadotropic hypogonadism, or smoking or alcohol consumption. Couples with previous ICSI attempts were excluded, and the cycles with fresh sperm were included.

A total of 540 couples meet the inclusion and exclusion criteria. We divided the population into three groups based on the maternal age to investigate the impact of maternal age on repairing sperm DNA damage and thus the ICSI outcomes. Sperm DNA fragmentation rates (%SDF) were assessed by the Sperm Chromatin Dispersion (SCD) method (Halosperm; Halotech, Spain). The groups were further divided into two groups based on the level of DNA fragmentation rates: the low (< 30% SDF) and high DNA fragmentation (≥ 30% SDF) groups. This SCD threshold has been associated with poor reproductive outcomes [18, 26, 27]. The outcome parameters were compared between the groups using generalized linear models followed by a Bonferroni post hoc test, with adjustment for potential confounders.

### Controlled Ovarian Stimulation (COS)

All women underwent controlled ovarian stimulation using either a short or long gonadotrophin-releasing hormone (GnRH) analog suppression protocol or a GnRH antagonist protocol and using recombinant follicle-stimulating hormone (Gonal-F, Merck Serono, Italy). Oocyte-retrieval was performed 35.5 h after the administration of 5000–10000 IU of human chorionic gonadotrophin (Ovitrelle, Merck Serono, Italy) to trigger the final follicular maturation when three or more follicles reached a diameter of ≥ 17 mm [28].

### Semen Analysis and Preparation

Semen samples were collected by masturbation after 3–5 days of sexual abstinence. After liquefaction, samples were analyzed according to the World Health Organization (WHO) guidelines [6]. Sperm morphology was assessed according to Kruger’s strict criteria after staining the semen smears with Diff-Quick (RAL Diagnostics, France) [29]. At least 100 sperm cells were counted, and normal morphology rates were expressed as % normal sperm morphology. Sperm samples were prepared using a two-layered (45%−90%) density gradient centrifugation (PureSperm, Nidacon) technique [30].

### Sperm DNA Fragmentation Assessment

Sperm DNA Fragmentation rates were assessed using the improved SCD test (Halosperm; Halotech, Spain) as previously described [26]. At least 100 sperm cells were examined, and the results were expressed as %DNA fragmentation rates.

### Intracytoplasmic Sperm Injection and Embryo Culture

Cumulus-oocyte complexes (COCs) were collected via a stereo microscope. Denudation of COCs was performed enzymatically by hyaluronidase (Hyase-10XTM, Vitrolife, Sweden) and by pipetting 4 h post-OPU. Maturity of the oocytes was assessed with X40 magnification. Oocytes with 1 st polar body (PB) were defined as mature oocytes (Metaphase II), and immature oocytes that extrude the first PB within 4 h were microinjected as described previously by Van Steirteghem et al., 1993 [31]. Injected oocytes were cultured individually in 25 mL microdrops of culture medium (IVF, Vitrolife, Sweden) covered with light paraffin oil (OVOIL, Vitrolife, Sweden) in 5% O2 chambers. Fertilization was checked after 16–18 h post-ICSI, and a healthy fertilization was observed by the presence of two pronuclei (PN) and two polar bodies (PB). Fertilization rates were expressed as %2PN and were calculated by dividing the number of fertilized oocytes by the number of mature oocytes. Embryonic development was assessed on days 2, 3, and 5, and embryo development rates were calculated by dividing the number of embryos by the number of mature oocytes. The quality of the embryos was also assessed with X40 magnification [32]. Grade A and B embryos were classified as good quality (4 cells on day 2/8–10 cells on day 3, < 15% fragmentation, symmetric cellular division, absence of multinucleation, cytoplasmic granulation, inclusion bodies, perivitelline space and zona pellucida abnormalities) and grade C and D embryos (the rest of embryos) were classified as poor quality. Blastocyst development was evaluated on the fifth day of development, according to Gardner’s classification, as full blastocysts, presenting separate and intact inner cell mass (ICM), presenting many cells and regular and tight trophectoderm, were defined as high-quality blastocysts, and the rest as poor-quality blastocysts [33].

### Embryo Transfer (ET) and Pregnancy Assessment

Embryo transfers were performed via ultrasonography on days 3–5, and one or a maximum of two embryos were transferred according to Turkish legal regulations. Serum β-hCG level was measured 12 days after the ET, and a ≥ 50 mIU/mL value indicated a positive pregnancy. Implantation rate (number of gestational sacs/number of embryos transferred) and clinical pregnancy rate (intrauterine ultrasonography with fetal heartbeat) were also assessed.

### Data Analysis and Statistics

The sample size calculation revealed that a sample size of 380 subjects had a 90% power to detect a 16% effect with a significance level of 5%. The calculations were done using G Power 3.1.7. ICSI parameters were compared between the groups using generalized linear models, followed by Bonferroni post-hoc via SPSS Statistics 21 (IBM). Data are expressed as mean ± standard error for continuous variables or percentages for dichotomous variables. A p-value of < 0.05 was considered statistically significant.

## Results

Demographic data and comparison of parameters among groups were demonstrated in Table 1. No significant differences were observed in laboratory and clinical outcomes, for cycles with either low or high SDF for the young group (≤ 36) and the group between 37–40 y.

**Table 1:** Relation between SDF and ICSI outcomes according to maternal age ranges (*n* = 540)

Variable	≤ 36 years old (*n* = 285)			37–40 years old (*n* = 147)			> 40 years old (*n* = 108)		
	< 30% SDF (*n* = 171)	≥ 30% SDF (*n* = 114)	*p* value	< 30% SDF (*n* = 99)	≥ 30% SDF (*n* = 48)	*p* value	< 30% SDF (*n* = 64)	≥ 30% SDF (*n* = 44)	*p* value
Paternal age (y)	32.1 ± 0.5	33.4 ± 1.1	.921	39.6 ± 1.9	42.3 ± 0.8	.233	45.3 ± 1.6	46.1 ± 0.9	.619
Sp concentration (mil/mL)	76.2 ± 18.3	89.4 ± 21.5	.689	69.7 ± 21.1	62.9 ± 17.9	.432	67.6 ± 35.5	86.5 ± 33.1	.562
Total motility (%)	66.1	72.6	.543	59.8	76.3	.042	58.4	65.5	.678
Progressive motility (%)	18.5	22.6	.455	16.2	19.3	.876	12.1	15.2	.394
Normal Morphology (%)	2.5	3.1	.566	3.2	2.9	.564	2.1	1.8	.912
Maternal age (y)	30.6 ± 1.1	31.5 ± 0.7	.573	38.3 ± 0.6	39.1 ± 0.7	.398	41.3 ± 2.1	42.1 ± 1.6	.713
Maternal BMI (kg/m2)	22.4 ± 0.4	21.9 ± 0.9	.467	23.1 ± 0.4	23.6 ± 1	.567	24.1 ± 0.7	23.4 ± 0.7	.754
Duration of ovarian stimulation (days)	9.3 ± 0.6	8.9 ± 1.2	.437	8.8 ± 1.1	9.1 ± 0.3	.655	9.1 ± 1.6	9.2 ± 2.2	.877
COC number (n)	16.2 ± 2.3	15.3 ± 1.6	.396	8.1 ± 2.6	8.7 ± 1.3	.417	4.1 ± 1.1	3.9 ± 2.9	.913
Mature oocytes (n)	13.1 ± 1.8	11.8 ± 1.4	.286	5.9 ± 2.1	5.1 ± 1.5	.545	3.0 ± 1.5	2.9 ± 2.6	.238
Fertilization rate (%)	88.3	86.7	.432	75.3	73.4	.659	68.3	66.6	.386
Embryo development rate (%)	75.2	77.5	.656	68.7	66.9	.766	57.5	59.6	.446
Blastocyst development rate (%)	49.9	48.5	.627	36.1	33.5	.488	29.4	23.8	.047
Good-quality embryos on D3 (%)	35.8	31.3	.865	28.6	27.1	.379	14.6	13.4	.042
Good-quality blastocyst rate on D5 (%)	45.3	40.2	.544	38.2	33.7	.557	20.2	16.6	.032
Number of Embryos transferred (n)	1.1 ± 0.1	1.3 ± 0.1	.654	2.0 ± 0.0	1.9 ± 0.1	.379	1.9 ± 0.1	1.7 ± 0.3	.916
Implantation rate (%)	35.6	31.5	.568	23.5	21.2	.333	12.4	10.6	.029
Pregnancy rate (%)	42.1	38.7	.567	31.7	26.9	.342	19.0	17.5	.039

Values are given as mean ± standard deviation, unless otherwise stated. *D3*, Day 3; *D5* Day 5; *FSH*, follicle stimulating hormone; *ICSI*, intracytoplasmic sperm injection; *SDF*, sperm DNA fragmentation

However, in the advanced maternal age group (> 40 y), lower embryo and blastocyst quality rates for both day 3 and 5, blastocyst development rate, and lower pregnancy and implantation rates were observed for cycles with high SDF compared with low SDF (Table 1).

## Discussion

DNA fragmentation of sperm is considered one of the major causes of male infertility and is influenced by several parameters, including oxidative stress, varicocele, and smoking etc. [34–36]. It was suggested to adversely impact reproductive outcomes regardless of other semen parameters, as well as the inheritance risk of paternal mutations [19, 37]. It is also reported to cause a decrease in the pregnancy rates and embryo quality in an IVF cycle [38].

One of the proposed mechanisms is that DNA fragmentation triggers apoptosis in sperm cells, which eventually eliminates sperm with damaged DNA, thus preventing the offspring [15]. But because apoptotic sperm cells display no visible signs until the end of the process, random selection of sperm cells according to only morphokinetic parameters during ICSI may adversely affect the outcomes. Although the DNA status of the injected sperm is not known, it is more likely to inject a DNA-damaged sperm in an infertile man in light of the data that DFI is higher in infertile men than fertile men [39].

Sperm cells are more prone to DNA damage than other cells, because they have/limited DNA repairing mechanisms [15, 22]. The suggested reasons include the tight packaging of sperm DNA by protamines and the limited amount of cytoplasm, which limits access of DNA repair factors to the damaged DNA [40, 41]. As a result, DNA damage is observed more frequently in sperm than in oocytes [42]. Repair of DNA damage in gametes and thus embryos is crucial for genomic stability and health of the offspring, since the sperm harboring DNA damage can still fertilize oocytes and develop into embryos.

Although sperm cells have no or limited chance to repair their genome, it is proposed that the oocytes may have the potential to compensate for the situation as the sperm enters the ooplasm [22]. However, it is technically and legally impossible to provide evidence for the human studies, and the studies can only focus on indirect estimations based on clinical outcomes. In light of the data that the cells’ repairing capacity decreases with advanced age, we investigated the role of maternal age on the damaged sperm DNA repairing capacity.

We demonstrated that in ICSI cycles with high sperm DNA fragmentation rates (SDF ≥ 30%), ICSI parameters of advanced maternal age (> 40), including embryo and blastocyst quality, blastocyst development, pregnancy, and implantation rates were decreased when compared to the younger patients. The results were similar in other maternal age and SDF groups. These findings are in accordance with the study, which reported that advanced maternal age is associated with reduced oocytes’ messenger RNA stores and thus DNA-repairing mechanisms [43].

The young and mid-aged women can repair sperm DNA damage, as demonstrated by the similar outcomes obtained in both low and high SDF groups.

The aged oocytes are solely known to affect the outcomes adversely because of increased incidence of aneuploidies [44], mitochondrial dysfunction [45–47], epigenetic regulations [48], meiotic spindle abnormalities [49, 50], meiotic segregation dysfunction [51, 52] and shortening of telomeres [53–55]. However, damaged sperm DNA would be an additional negative factor on these adverse effects, which makes up 50% of the embryonic genome. This suggestion is proven by the findings of the control group, which has similar demographics, including the advanced maternal age, and the only difference between the groups was the sperm DNA fragmentation index.

Some of the proposed mechanisms underlying the situation include age-related decline of the BRCA-related ATM-mediated DNA repair pathway [24], DNA base-excision repair (BER) pathway proteins, including APE1, PARP1, XRCC1, Polβ (POLB), and LIG3, which is responsible for single-strand DNA (ssDNA) repair [25]. A recent systematic review [22] reported that the early embryo can repair DNA damage within sperm by activating maternally driven mechanisms during embryonic development in both humans and animals. This capacity is reported to be limited and likely to decline with age. This is explained by a decrease in the mRNA of key DNA repair and DNA damage response genes observed in aging oocytes [43, 56–59]. But it is concluded that, because there is not always a correlation between mRNA levels and their protein products, further studies are needed to investigate the protein expression in oocytes and early embryos to examine if there is a decrease in the DNA repair machinery with age. In an animal study, 91 DNA repair genes were investigated, and reduced expression for 23 genes was observed in older oocytes. These genes affected pathways, including double-strand break, nucleotide excision repair, and DNA damage response [59]. In other animal studies, it has been shown that the oocyte can initiate repair of DNA strand breaks before the first cleavage in early embryo development in the mouse [60–62]. And thus suggested that oocyte DNA repair capacity could influence the clinical effects of sperm DNA damage in ART.

The present study has several limitations, including our inability to directly test DNA repair mechanisms in oocytes or embryos because of legal regulations and ethical concerns. However, increasing the number of studies and the number of subjects will contribute to making the results more reliable and enlightening the issue. Our study contributes to the present knowledge with a relatively larger sample size and additional outcome measure parameters. Another limitation is that only a single method is used to determine SDF since the technique is routinely used as a part of all IVF cycles. Although the technique is reliable and commonly used, it would be more reliable to corroborate the results with other methods.

As a conclusion, it may be suggested that older oocytes seem to have a lower potential to repair sperm DNA damage based on our results, which demonstrate decreased IVF outcome parameters in couples with higher SDF scores, if maternal age is higher (≥ 40).

Novel sperm selection methods, such as APB-Tech [63], hyaluronic acid binding [64], magnetic sorting [65], and high magnification microscopy [66], seem to isolate less damaged spermatozoa, thus may have the potential to decrease the aforementioned adverse effects.

Finally, understanding the molecular mechanisms of the repairing capacity of oocytes and embryos is of great importance to fully elucidate the topic, thus, further investigation into these mechanisms will aid in developing treatment strategies to improve and preserve fertility in advanced reproductive age.

## Data Availability

Not applicable.
